# Carbonization and H_3_PO_4_ activation of fern *Dicranopteris linearis* and electrochemical properties for electric double layer capacitor electrode

**DOI:** 10.1038/s41598-020-77099-7

**Published:** 2020-11-17

**Authors:** Trang K. Trinh, Toshiki Tsubota, Shuto Takahashi, Nga T. Mai, Minh N. Nguyen, Nam H. Nguyen

**Affiliations:** 1 Department of Materials Science, Faculty of Engineering, Kyushu Institute of Technology, 1-1 Sensuicho, Tobata-ku, Kitakyushu, Fukuoka 804-8550 Japan; 2 Faculty of Environmental and Natural Resources, Ha Tay Community College, Thuy Xuan Tien Ward, Chuong My District, Hanoi, Vietnam; 3 Faculty of Environmental Sciences, University of Science, Vietnam National University, Hanoi (VNU), 334 Nguyen Trai, Thanh Xuan, Hanoi, Vietnam; 4 Energy Department, University of Science and Technology of Hanoi, Vietnam Academy of Science and Technology, 18 Hoang Quoc Viet Street, Cau Giay District, Hanoi, Vietnam

**Keywords:** Environmental sciences, Materials science

## Abstract

Today, the world’s climate change is a growing problem, plant carbon sequestration is one of the effective ways to mitigate climate change by reducing greenhouse gases, mostly carbon gases. *Dicranopteris linearis* (*D. linearis*), a common fern species in the tropic or subtropic ecoregions, has been recently recognized as a potential feedstock to produce highly porous biochar. This study aims to enhance the specific surface area (SSA) and pore volumes of biochars derived from the *D. linearis* by H_3_PO_4_ activation and examine electrical properties of the activated biochars and their possible usage for the electric double-layer capacitor (EDLC) electrode. The treated raw fern was activated with H_3_PO_4_ 85% by the three different mixing ratios 1:0, 1:1, and 1:3 (w/w) and then pyrolysis under N_2_ flow maintained at 500 °C for 1 h. The performance as the electrode for an EDLC was evaluated in 1 mol L^−1^ H_2_SO_4_ solution for the H_3_PO_4_-activated samples. The SSA and pore volumes were drastically increased after activation. The maximum SSA and pore volume were 1212 m^2^ g^−1^ and 1.43 cm^3^ g^−1^, respectively for the biochar activated at 400 °C with a weight mixing ratio 1:3 (w/w) between the fern and H_3_PO_4_ acid while these values of the biochar at 400 °C were 12 m^2^ g^−1^ and 0.02 cm^3^ g^−1^, respectively. The biochar activated at 600 °C with the mixing ratio 1:1 (w/w) showed the maximum capacitance value, *ca*. 108 F g^−1^ at 1 mV s^−1^. The activation using H_3_PO_4_ showed a positive tendency to enhance electrochemical properties and it could be a premise toward a higher performance of EDLC from the *D. linearis* derived activated biochar.

## Introduction

Today, the world’s climate change is a growing problem, increasing ocean depth, changing plant and animal habitats, and making storm stronger and more dangerous^1^. Climate change is caused by greenhouse gases, mostly carbon gases that build up in our atmosphere trapping the heat from the sun and stopping it from escaping into space. Carbon gases such CO_2_ and CO are mainly released when people use fossil fuels such as gas diesel and coal. It is important to take carbon out of the air to reduce the impact of greenhouse effects. The process of removing carbon from the air and storing is called carbon sequestration. One effective and natural method of carbon sequestration that tree suck in carbon and converted into wood which they bury deep underground in the form of roots which can be turn into charcoal by controlled or naturally burns. However, one major challenge of using biomass as an energy carrier is the treatment of such emissions during the thermal process (combustion/pyrolysis). Nitrogen oxides (NO*x*), are considered one of the main gas emissions during the thermal processes of biomass and NO*x* family gases are often recognized to cause acid rain^2^. Therefore, these kinds of emissions need to be controlled to reduce their impact on the environment and human health^3^. Hence, there are more and more researchers use biomass as a precursor material for producing biochars or activated biochars in controlled conditions.

Fern *Dicranopteris linearis* (*D. linearis*) is known as one of the most serious weed species due to their widespread from tropical to subtropical ecoregions^4^. Nevertheless, their economic value has received little attention. In many remote regions, this fern is burned to fertile soils (slash-and-burn agriculture) or used as a material for covering soil surface or earlier it has been an important fuel for local minorities. More recently, *D. linearis* has been more intensively studied and the findings suggested this fern species as a potential material for biochar production^5^ or soil improvement^6,7^. This encourages more research works to get the advantages from this type of species economically and efficiently^5–10^.

Activated carbon has a wide range of applications in reality such as medicine, industry, agriculture, and the environment due to its ability of adsorption and desorption. Activated carbon is known as purified charcoal, treated physically or chemically to generated micro-fissures that vastly increase its SSA, thereby enhancing exposure for adsorption or chemical reactions^11^. Chemical activation usually provides activated carbon with higher SSA than that of activated carbon prepared by physical activation. Chemical substance as phosphoric acid (H_3_PO_4_) is a highly recommended activator for the preparation of activated carbon due to its benefits of relatively lower activation temperature than other typical alkali activators such as KOH, K_2_CO_3_, NaOH, etc. For example, Hayashi et al., reported that in comparison with other alkali activators, H_3_PO_4_ allowed to obtain the high SSA (> 1000 m^2^ g^−1^) and total pore volumes at the lower temperature range (400–600 °C) while the effective temperature for alkali activators was 800 °C^12^. The lower temperature activation means safer, more economical, and more convenient for practical uses. Moreover, H_3_PO_4_ has a relatively lower toxicity than other common activators such as ZnCl_2_ and KOH. Many researchers have paid attention to H_3_PO_4_ activation to enhance SSA and total pore volumes of their carbon material^12–18^. Abdenaeim and De Yuso reported that the main mechanisms of activation with phosphoric acid are the depolymerization, dehydration and redistribution of biopolymers in lignocellulosic materials^19,20^ to created tunnel-shaped and generally have a honeycomb structure on the surface of activated carbon^21,22^. Activated carbon with large SSA and suitable pore size, which also leads to high electrical conductivity and enhance the performance of the electric double-layer capacitor (EDLC) electrode, a well-known electric power storage device nowadays^23–25^.

The electric power storage in EDLC is performed by utilizing the interlayer between electrode surfaces and electrolytes. So far, many kinds of agricultural wastes such as pine cones, coconuts shells, rice husk^26^, strobili fibers^27^, durian shells^28^, bamboos^29^, starch^30^ and flowers^31^ have been investigated as promising raw materials to create activated carbon for the EDLC electrode. Having higher SSA in comparison with other biochar prepared at the same pyrolysis condition^5^, fern *D. linearis* can also be a promising material to prepared activated biochar and use for the EDLC electrode. This study evaluated the performance of H_3_PO_4_ in activating fern *D. linearis* derived biochars, electrical properties of the activated biochars and the feasibility of using these activated materials for well-performed EDLC electrodes.

## Material and methods

### Material and sample preparation

#### Production of biochars

The fern *D. linearis* was collected in a hilly area at Pho Yen district, Thai Nguyen province, Vietnam (Long: 105.485° E; Lat: 22.762° N) was air-dried, chopped into small segments (size ~ 1 mm) and then kept in the dried and clean plastic bottles for further investigation. Detailed description of the heat treatment apparatus used in this study is shown in Supplementary Fig. S1 online. Pyrolysis of the *D. linearis* biomass was conducted under N_2_-supported at three different temperatures, i.e., 400, 600 and 800 °C in an oven (Nabertherm LT 24/12/P300). For each experiment, approximately 300 g of D. *linearis* was put into an airtight steel box (10 cm in height and 20 cm in diameter). The pyrolysis process was installed at a heating rate of 15 °C min^−1^ with N_2_ supporting at 3 NL min^−1^ (NL: Normal litter at 273 K and 1 atm) and maintained for 1 h.

#### Activation procedure

For activation, each 60 g of D. *linearis* biomass was mixed with H_3_PO_4_ (85%) by the mixing ratio 1:0, 1:1, and 1:3 (w/w) in an airtight refractory steel box 25 cm in diameter and 20 cm in height and put in an electric muffle furnace (Nabertherm LT 24/12/P300). The pyrolysis process was installed at a heating rate of 15 °C min^−1^ with N_2_ supporting at 3 NL min^−1^ (NL: Normal litter at 273 K and 1 atm) and maintained at 500 °C for 1 h.

### Characterizations

Chemical composition of *D. linearis* biomass was examined by executing wet digestion with aqua regia solution and then dissolved Si was determined by molybdate blue method while other dissolved ions were analyzed by inductively coupled plasma mass spectrometry (ICP-MS, Agilent 7900). The micro-structures of the samples were observed by scanning electron microscope (SEM, JEM5310, JEOL). To characterize the SSA and pore volumes, N_2_ adsorption isotherms and CO_2_ adsorption isotherms were measured by a commercial apparatus (BELSORP mini II, MicrotracBEL Corporation instrument). The data of N_2_ adsorption isotherms were used for the estimation of Barrett–Brunauer–Emmett–Teller (BET) SSA, total pore volume, and micro-pore volume by using the *t*-plot statistical thickness method. CO_2_ adsorption isotherms were used for the estimation of micro-pore volume by using Dubinin–Astakhov (DA) method. To understand the properties of the samples, several methods were conducted such as CHN measurement (CHN corder MT-5, Yanako), XRF measurement (S8 TIGER, Bruker), and ^13^C NMR measurement (JNM-ECX400, JEOL) for understanding the kind of elements in the samples. The measurement conditions of ^13^C NMR were as follows, DEPTH2, CPMAS probe: 3.2 mm, MAS (Magic Angle Spinning) speed: 15 kHz. Moreover, FT-IR (Nicolet 6700, Thermo scientific) (ATR method) and XPS (AXIS-NOVA, Shimadzu/KRATOS) for defining the chemicals bonds and functional groups.

### Electrochemical properties

Evaluation of the electrochemical performance as an electrode of electric double-layer capacitor was performed following the procedure suggested by Tsubota et al.^29–34^. The activated biochars were mixed with polytetrafluoroethylene (PTFE) and acetylene black (AB) by the mixing ratio 8:1:1 (w/w/w). PTFE played a role as a binder, while acetylene black (AB) acted as conductive agent. The mixtures were rolled to *ca*. 0.5 mm thickness, and then were cut into the rectangle shape 20 mm × 8 mm samples. The sheet samples were set on a Pt plate for collecting electrode, and a three-electrode cell was prepared for the electrochemical measurements. A 1 M H_2_SO_4_ aqueous solution, a Pt plate, and an Ag/AgCl electrode were used as electrolyte, a counter electrode, and reference electrode, respectively. The electrochemical measurements, such as cyclic voltammetry (CV) and galvanostatic charge–discharge measurement, were performed for the evaluation of electrochemical performance.

## Results and discussion

### Raw material

The elements in raw fern D. *linearis* were shown in Supplementary Fig. S2 online. The result shown that K, Na, Ca, Al, Si are the main inorganic elements in the chemical composition of the D. linearis fern. Alkaline metal (K, Na) and alkaline-earth metal (Ca, Mg) were main elements, but Al and Si also existed in the fern. All toxic heavy metals as Cr, Ni, As, Cd, Hg, Pb, etc. did not appear so this demonstrated the purity of raw material.

### Characteristics of biochars and activated biochars

**Figure 1:**
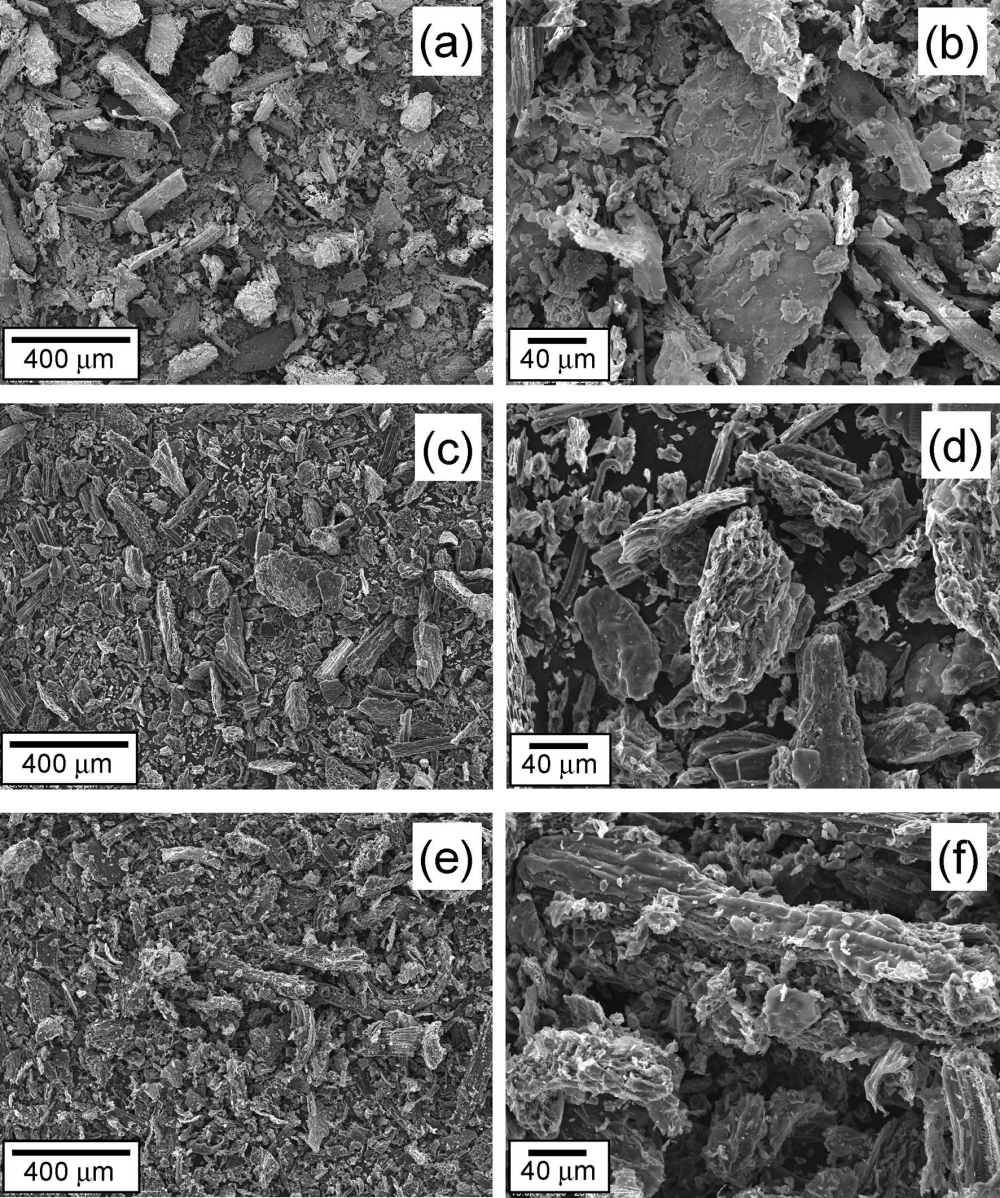
SEM images of the samples. (**a**,**b**) Carbonized at 400 °C, (**c**,**d**) Carbonized at 400 °C, (**e**,**f**) Carbonized at 400 °C.

The SEM (Fig. 1) images of the carbonized samples indicated that the sizes of the particles, which had the micro-structure derived from plant issues, were independent of the carbonization temperature. Therefore, the structure of raw material was not destroyed by the carbonization at the temperature region (400–800 °C).

**Figure 2:**
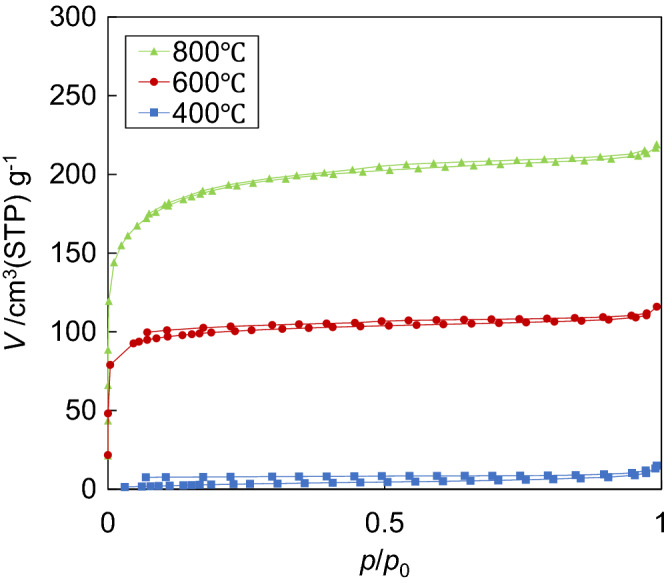
N_2_ adsorption isotherms of the samples.

The N_2_ adsorption isotherms at 77 K of the carbonized samples are shown in Fig. 2. The adsorbed volumes of the sample carbonized at 400 °C were relatively low, while these volumes of biochars obtained at 600 °C and 800 °C were obviously higher; and increased rapidly in the region of the low relative pressure. In addition, the curves of all samples did not close in the low-pressure region, which was considered as low-pressure hysteresis. The low-pressure hysteresis appeared when the diameters of pores were near to the size of nitrogen molecule (approximate 0.35 nm). The difference between adsorption isotherm and desorption isotherm at low pressure decreased when the pyrolysis temperatures increased.

The CO_2_ adsorption isotherms at 298 K of the carbonized samples are shown in Supplementary Fig. S3 online. The patterns of the adsorbed volumes obtained from the biochars carbonized at 600 °C and 800 °C were similar. These volumes are much larger than that volume of the 400 °C sample. The drastic increment of the adsorbed volumes from 400 to 600 °C means that the volume of pore less than 0.7 nm in diameter classified as ultra-micropores largely increased from 400 to 600 °C. The chemical reactions for carbonization, such as pyrolysis, generate gaseous species leading to the formation of ultra-micropores progress in the region from 400 to 600 °C; and the irradiations of these gases increased significantly from 400 to 600 °C. The slight increment of adsorbed volumes from 600 to 800 °C could be reasonable due to the carbonization reactions which generate gaseous molecules until 600 °C.

The values related to SSA and pore volume estimated from N_2_ adsorption isotherms and CO_2_ adsorption isotherms are listed in Table 1. The BET SSA (*S*_BET_), total pore volume (*V*_total_), and micropore volume (*V*_micro_) estimated from CO_2_ adsorption isotherms by Dubinin-Astakhov (DA) model increased with increasing the carbonization temperature. The index number of DA models depends on the pyrolysis temperature and relates to the pore size distribution, so pyrolysis temperatures should affect the pore size distribution of the samples. The change in SSA and the pore conditions such as pore volumes and pore size distribution of the samples as the follows.

- At 400 °C, pores were poorly generated. Most of the pores had very small diameters, which cause low-pressure hysteresis at N_2_ adsorption isotherm.
- At 600 °C, relatively many pores were created by the irradiation of gaseous molecules derived during the pyrolysis processes. Therefore, the volume of ultra-micropore and micropore significantly increased for both N_2_ and CO_2_ adsorption isotherms when pyrolysis temperature changed from 400 to 600 °C.
- At 800 °C, the diameter of pores increased, which was explained by the increase of the N_2_ adsorbed volumes from 600 to 800 °C as shown in Fig. 2. The N_2_ adsorbed volumes at 800 °C were about two times higher than the value at 600 °C, while in CO_2_ adsorption isotherm these values were similar at both temperatures. The larger molecules are diffused into the larger pores on the sample surfaces, so the discrepancy of N_2_ adsorbed volumes at 800 °C and 600 °C indicated the difference of pore size. In that case, samples prepared at 800 °C has larger pore size compare to others.

**Table 1:** The values estimated from adsorption isotherms.

	*S*_BET_ (m^2^ g^−1^)	*V*_total_ (cm^3^ g^−1^)	*V*_micro_(DA) (cm^3^ g^−1^)	Index number (of DA)
400 ℃	12	0.02	0.13	2.3
600 ℃	387	0.18	0.27	1.9
800 ℃	702	0.34	0.39	1.7

The contents of C, H and N are listed in Table 2. The ash content was 9.74 wt.%. The XRF result illustrated that the ash contents of the sample mainly consist of K, Ca, Si, and Al in Table 3. In comparison with the chemical components of raw material, Na no longer appeared in the biochar samples. That means Na elements were removed during the pyrolysis process. Nguyen et al.^35^ reported that the relationship of pyrolysis temperature and potassium (K) which element has the highest percentage in the XRF detection. From XPS data Nguyen et al. found that the intensities of the peaks related to K decreased with increasing the heating temperature and disappeared at 800 °C so the K atoms changed to higher valence at 600 °C^35^.

**Table 2:** CHN measurement for G600.

Element	wt.%
C	75.61
H	2.34
N	1.50
Residue	9.74
Others	10.81

**Table 3:** XRF result for G600.

Element	wt.%
K	31.41
Ca	27.44
Si	20.88
Al	8.84
P	3.19
Fe	2.64
Mg	1.89
Mn	1.52
S	1.20
Cl	0.99

### H_3_PO_4_ activation

The SEM images (see Supplementary Fig. S4 online) indicated the micro-structure of the H_3_PO_4_-activated biochars in which the particles were independent of the activation condition such as temperature and the amount of added H_3_PO_4_. Some parts of the activated biochars were likely slagged leading to smoother surface and unified pores.

**Figure 3:**
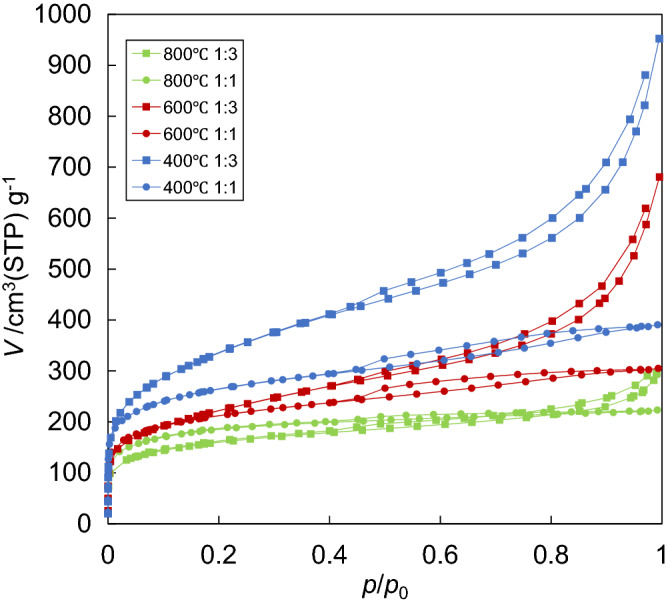
N_2_ adsorption isotherms of the samples activated with H_3_PO_4_.

The N_2_ adsorption isotherms of the activated samples are shown in Fig. 3. The adsorbed volume increased with the increasing amount of H_3_PO_4_ and decreased with increasing the heating temperature. When the mixing ratio of H_3_PO_4_ to raw material was 3, the adsorbed volume rapidly increased with the relative pressure (*p*/*p*_0_) higher than 0.8. Because the size distributions of the activated biochars were similar each other, the origin of the rapid increment was defined by the increase of N_2_ diffusion not into the interparticle void but into the pores on particles. Luo et al. also reported^17^ that the SSA and total pore volume dramatically increased with the mixing ratio 1:3 (w/w), and then decreased in the higher mixing ratio. The reason for the decrement was presumed by Luo et al.^17^ that higher concentration of H_3_PO_4_ forms some phosphorus compounds which blocked the porosity of activated carbon. The N_2_ adsorption isotherms for H_3_PO_4_ activation reported by Luo et al.^17^ and by Hayashi et al.^12^ were different from those of this study. They reported that pore volume and SSA decreased with increasing the activation temperature at > 600 °C while this study showed the highest values at 600 °C and then decreased at the higher pyrolysis temperatures. The differences in pretreatment processes and raw biomass sources were the reasons for the distinctive results. Figure 3 showed the hysteresis loop which closed around 0.4 of *p*/*p*_0_ explained by the theory based on capillary condensation in pores. Because capillary condensation can occur in mesopore (2–50 nm), these samples should have mesopores.

The CO_2_ adsorption isotherms at 298 K are shown in Supplementary Fig. S5 online. The adsorbed CO_2_ volume decreased with increasing the added H_3_PO_4_ amount. Because the adsorbed CO_2_ volume should be related to ultra-micropore (< 0.7 nm in diameter), the decrease of the adsorbed volume should mean the reduction of the volume for very small pores such as ultra-micropore of the sample. One of the reasons lead to the decrements is the expansion of pore sizes (larger than CO_2_ molecule) due to the superimposing of ultra-micropores in the biochars activated by adding a higher H_3_PO_4_ amount.

The values related to SSA and pore volumes were estimated these adsorption isotherms and were listed in Table 4. The curves in Fig. 3 represent type IV adsorption isotherm. The vertical increment at the near-zero of relative pressures should indicate the existence of micropores. Because all the values of *V*_micro_ were estimated from N_2_ adsorption isotherms by using the *t*-plot theory, the estimated pore volume roughly assigned to 0.7–2.0 nm in diameter. *V*_micro_ (DA) was estimated from CO_2_ adsorption isotherm with extrapolation. *V*_meso_ was estimated from N_2_ adsorption isotherm by using the BJH theory. The sample with the mixing ratio 1:3 (w/w) prepared at 400 °C had the largest *S*_BET_ value (1212 m^2^ g^−1^) and all the volumes. In comparison with other biomass sources in Supplementary Table S1 online, the *S*_BET_ of D. *linearis* activated biochar is similar while *V*_total_ and *V*_micro_ are higher than most of others^15,16,28,36–42^. Therefore, fern D. *linearis* derived activated carbon with both micro and mesopores could be effectively prepared by H_3_PO_4_ activation.

**Table 4:** The values estimated from adsorption isotherms for the samples activated with H_3_PO_4_.

H_3_PO_4_ activation	*S*_BET_ (m^2^ g^−1^)	*V*_total_ (cm^3^ g^−1^)	*V*_micro_ (cm^3^ g^−1^)	*V*_meso_ (cm^3^ g^−1^)	*V*_micro_(DA) (cm^3^ g^−1^)	Index number (of DA)
400 °C 1:1	944	0.60	0.39	0.31	0.42	1.7
400 °C 1:3	1212	1.43	1.32	1.20	0.58	1.4
600 °C 1:1	758	0.47	0.47	0.24	0.34	1.7
600 °C 1:3	761	0.92	0.90	0.78	0.38	1.5
800 °C 1:1	660	0.35	0.34	0.11	0.32	1.7
800 °C 1:3	570	0.46	0.39	0.28	0.24	1.7

It is obvious from the experimental results of N_2_ and CO_2_ adsorption isotherms listed in Tables 1 and 4 that both SSA and total pore volume were significantly enhanced by H_3_PO_4_ activation. Processes that occurred during activation with H_3_PO_4_ acid were the cellulose depolymerization, biopolymers dehydration, and redistribution of biopolymers in biomass^43,44^, which enhanced the total pore volumes and SSA. The H_3_PO_4_ added in the raw material acted as the catalyst for the cleavage of cellulose fibers and cross-links in the biomass shell to formation of gases, volatile fragments and soluble products during the activation processes^28,44,45^. In Supplementary Table S2 online, The C contents of the samples prepared by H_3_PO_4_ activation were lower than those of the samples that were simply prepared by carbonization, while the other remaining compounds increased with increasing the amount of added H_3_PO_4._ The reason could be presumed that the appearance of some inorganic compounds generated during H_3_PO_4_ activation such as polyphosphoric acid. The XPS (in Supplementary Fig. S6 online) demonstrated the intensive of others element like N, P, Si, etc., increase with the increase of temperature and the H_3_PO_4_ amount. ^13^C NMR graphs shown in the Supplementary Fig. S7 online has been shown that the activated carbon prepared with phosphoric acid has less C=O groups than the raw materials. The reduction of carbonyl groups may be due to the effect of H_3_PO_4_ hydrolysis, which decomposes these groups and other products such as volatile substances^46^. The FT-IR chart show (Supplementary Fig. S8 online) that the transmittance of O–H and C=O group decrease with the increase of temperature and H_3_PO_4_ doses. These experimental results indicate that the addition of H_3_PO_4_ affects the functional groups in biochar.

### Electrochemical properties

The results of cyclic voltammetry (CV) (scan rate: 1 mV s^−1^) are shown in Supplementary Fig. S9 online. The larger area of CV graph indicates the capacitance value, and the like-rectangular shapes of CV graph show the capacitance derived from electric double layer. Therefore, the large area with rectangular shape of CV graph means the high performance of electrochemical properties of the sample. When the activation temperature was 600 °C, the CV graph of the sample tended to a larger area than that of others. The trend of the activated sample prepared at 600 °C were relatively rectangular although some peaks were still observed. Despite having larger SSA and pore volumes, the activated carbons prepared at 400 °C revealed their smaller areas of CV charts as compare to the 600 °C activated samples. In general, electrical conductivity depends on the treated temperature. The electrical conductivity of carbonized samples drastically increases from about 800 to c.a. 1000 °C. Electrical conductivity is one of the important factors which affect the capacitance values. At lower treated temperature (e.g., 400 °C) can be the reason why activated carbons at 400 °C has low capacitance while its SSA is the highest.

The pyrolysis temperatures dependence of the capacitance values at 1 mV s^−1^ are shown in Supplementary Fig. S10 online. The samples prepared with the mixing ratio 1:1 (w/w) had the higher capacitance values than the samples prepared with the mixing ratio 1:3 at all carbonization temperature. It was likely that H_3_PO_4_ activation were effective at the low impregnation ratio for large capacitance value. The presumed reason could be excessive phosphoric acids reacted more with organic compounds in the precursor leading to the formation of phosphate and polyphosphate bridge and esters linkage with OH^−^ group at cellulose^44^ as electrical insulating layers. The sample prepared at 600 °C with the mixing ratio 1:1 (w/w) had the highest value of capacitance about 110 F g^−1^ (scan rate 1 mV s^−1^). The capacitance values are higher than the previously reported values such as 82.5 F g^−1^ (scan rate: 50 mV s^−1^) and 72 F g^−1^ (scan rate: 200 mV s^−1^) of activated biochar derived from durian prepared by H_3_PO_4_ activation^28^. However, the activated carbons prepared in this study was not considered as high electrochemical performances because the capacitance value per weight estimated by two-electrode cell is 1/4 times in comparison with 3-electrode system.

**Figure 4:**
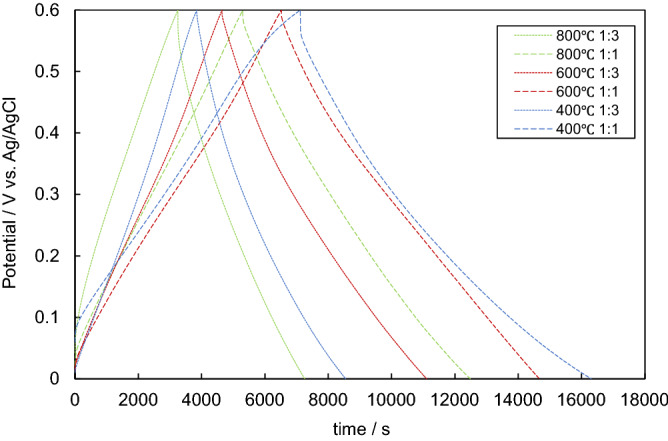
The graphs of charge–discharge measurement at 10 mA g^−1^ of the samples activated with H_3_PO_4_.

The patterns of charge–discharge measurement at 10 mA g^−1^ are shown in Fig. 4. The shapes of these graphs were nearly isosceles triangles indicating that the electric energy was stored not by redox reactions but by the electric double layer. The shape type of charge and discharge curve is usually obtained from the carbon-based electrode EDLC^28,31^. Therefore, the samples in the cells act as the active material of EDLC.

The capacitance values calculated from the results of charge–discharge measurements are shown in Supplementary Fig. S11 online. Recent studies have reported the capacitance values which were measured in the electrolyte of 1 M H_2_SO_4_ aqueous solution^31,47,48^. One of the reasons for lower capacitance values at higher current densities could be the low electrical conductivity of the activated samples. Moreover, the lower capacitance values also attributed to insufficient electrolyte ions diffusion kinetics across the micropores. The reduced accumulation amount of electrolyte ions onto porous electrode interfaced results of the reduction in specific capacitance^31^. The specific capacitance values of all the activated biochars of this study nearly closed to 0 at 1000 mA g^−1^, which is not suitable as an EDLC electrode. Chang et al.^31^ reported an effective result that the capacitance value of 211.6 F g^−1^ could remain at 10,000 mA g^−1^, revealing that the large capacitance value at high current density region. In spite that some of the activated biochars in the study had mesopores, the capacitance values of the samples were still low at high current density regions. The explanation is low electrical conductivity of the carbonized structure and to exist electrical insulators formed by the components generated during activation like polyphosphoric acid, oxides, and oxygen-containing functional groups as Molina-Sabio and Rodríguez-Reinoso recorded in his study^44^. Reducing the electrical resistance or remove the insulators layers is effective to improve the performance of EDLC electrodes, which might be interesting idea for future studies.

## Conclusions

The present work focused on developing the SSA and total pore volume of biochar derived from fern D. *linearis* by H_3_PO_4_ activation and check the possibility of electrochemical performance for EDLC electrodes. All the N_2_ adsorption isotherms for the biochars prepared at 400–800 °C showed low-pressure hysteresis, which indicates the existence of ultra-micropores. Dominant elements in the sample carbonized at 600 °C were potassium and calcium except for the elements related to organic compounds, such as carbon, oxygen, hydrogen. The activation by H_3_PO_4_ was effective for enhancing both the SSA and pore volume. The sample activated at 400 °C with the mixing ratio 1:3 (w/w) had the maximum values. The maximum capacitance value was ca. 108 F g^−1^ at 1 mV s^−1^ for the sample activated at 600 °C with the mixing ratio 1:1 (w/w) at the weight. The research opened an idea of a new research about suppression of electrical resistance to improve of the electrochemical performance.

## Supplementary information

Supplementary Tables.

Supplementary Figures.
