# Supplementary material for: Carbonization and H3PO4 activation of fern Dicranopteris linearis and electrochemical properties for electric double layer capacitor electrode
Source: Sci Rep. 2020 Nov 17;10:19974. doi: 10.1038/s41598-020-77099-7 (PMC7672103; doi:10.1038/s41598-020-77099-7)
Supplement: Supplementary file 1 — Supplementary Tables. [file 41598_2020_77099_MOESM1_ESM.pptx]

## Slide 1
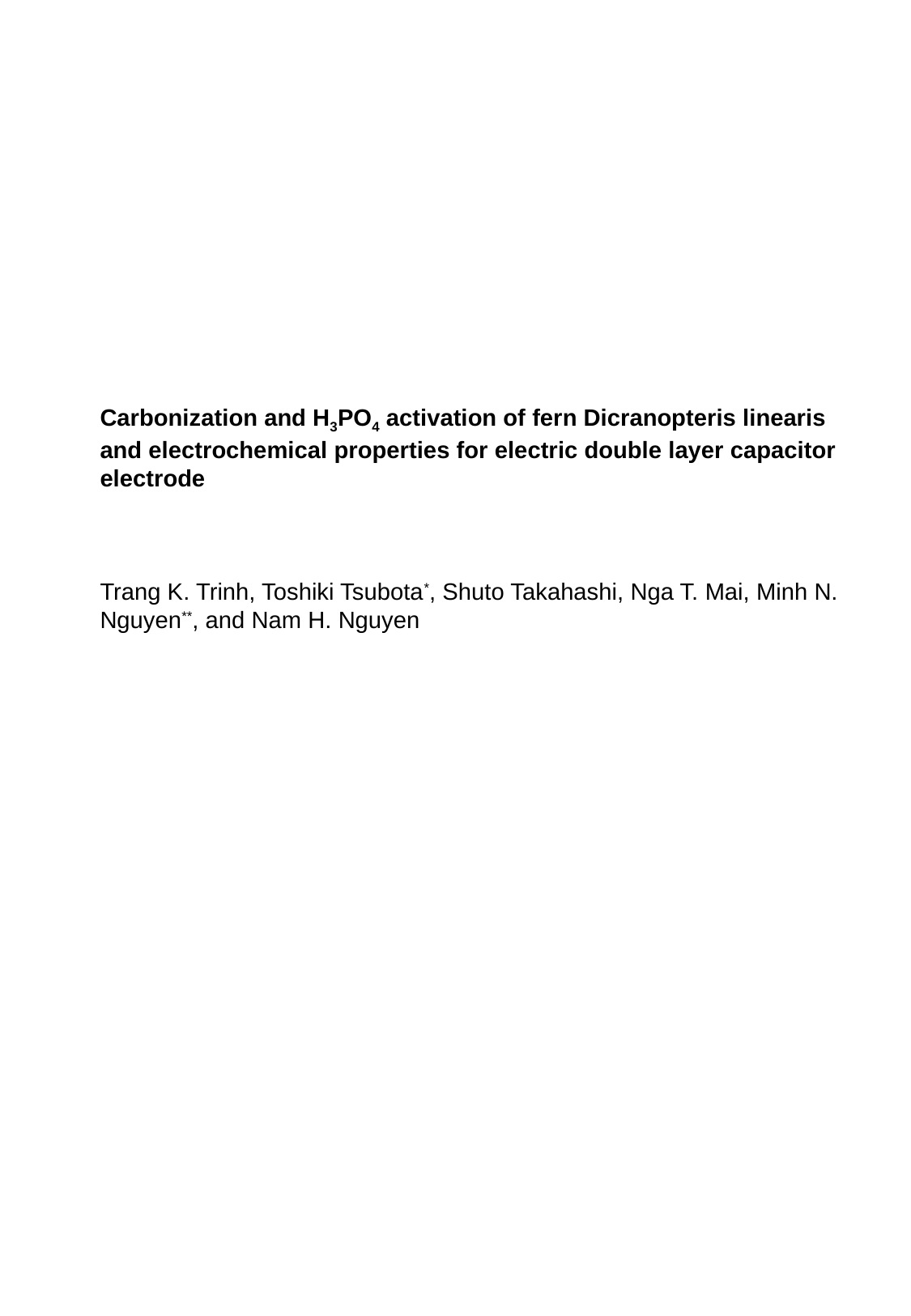

Carbonization and H3PO4 activation of fern Dicranopteris linearis and electrochemical properties for electric double layer capacitor electrode
Trang K. Trinh, Toshiki Tsubota*, Shuto Takahashi, Nga T. Mai, Minh N. Nguyen**, and Nam H. Nguyen

## Slide 2
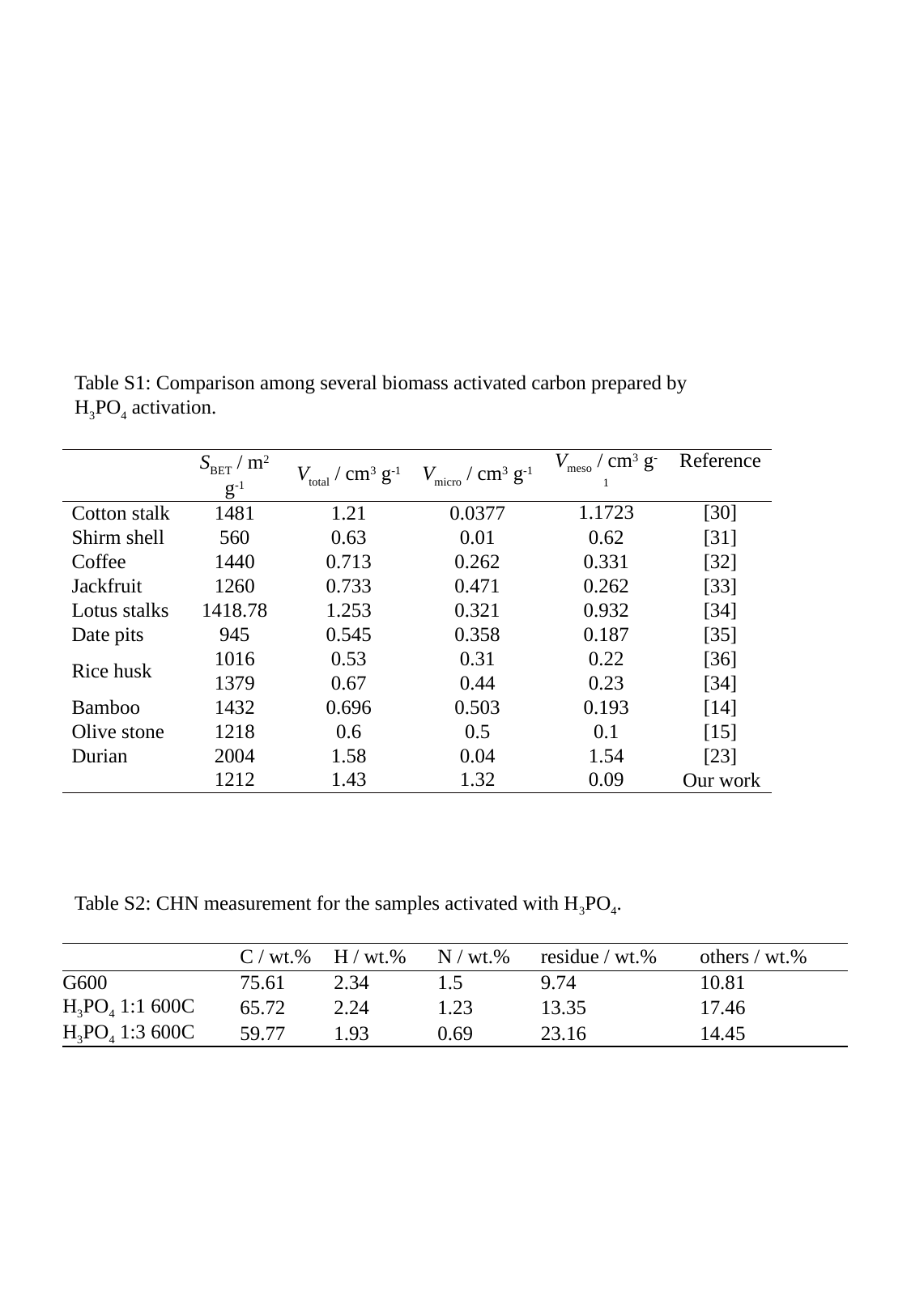

Table S1: Comparison among several biomass activated carbon prepared by H3PO4 activation.
| | SBET / m2 g-1 | Vtotal / cm3 g-1 | Vmicro / cm3 g-1 | Vmeso / cm3 g-1 | Reference |
| --- | --- | --- | --- | --- | --- |
| Cotton stalk | 1481 | 1.21 | 0.0377 | 1.1723 | [30] |
| Shirm shell | 560 | 0.63 | 0.01 | 0.62 | [31] |
| Coffee | 1440 | 0.713 | 0.262 | 0.331 | [32] |
| Jackfruit | 1260 | 0.733 | 0.471 | 0.262 | [33] |
| Lotus stalks | 1418.78 | 1.253 | 0.321 | 0.932 | [34] |
| Date pits | 945 | 0.545 | 0.358 | 0.187 | [35] |
| Rice husk | 1016 | 0.53 | 0.31 | 0.22 | [36] |
| | 1379 | 0.67 | 0.44 | 0.23 | [34] |
| Bamboo | 1432 | 0.696 | 0.503 | 0.193 | [14] |
| Olive stone | 1218 | 0.6 | 0.5 | 0.1 | [15] |
| Durian | 2004 | 1.58 | 0.04 | 1.54 | [23] |
| | 1212 | 1.43 | 1.32 | 0.09 | Our work |
Table S2: CHN measurement for the samples activated with H3PO4.
| | C / wt.% | H / wt.% | N / wt.% | residue / wt.% | others / wt.% |
| --- | --- | --- | --- | --- | --- |
| G600 | 75.61 | 2.34 | 1.5 | 9.74 | 10.81 |
| H3PO4 1:1 600C | 65.72 | 2.24 | 1.23 | 13.35 | 17.46 |
| H3PO4 1:3 600C | 59.77 | 1.93 | 0.69 | 23.16 | 14.45 |
