# Supplementary material for: Carbonization and H3PO4 activation of fern Dicranopteris linearis and electrochemical properties for electric double layer capacitor electrode
Source: Sci Rep. 2020 Nov 17;10:19974. doi: 10.1038/s41598-020-77099-7 (PMC7672103; doi:10.1038/s41598-020-77099-7)
Supplement: Supplementary file 2 — Supplementary Figures. [file 41598_2020_77099_MOESM2_ESM.pdf]

Carbonization and H_3PO_4 activation of fern Dicranopteris linearis and electrochemical properties for electric double layer capacitor electrode

Trang K. Trinh, Toshiki Tsubota*, Shuto Takahashi, Nga T. Mai, Minh N. Nguyen**, and Nam H. Nguyen

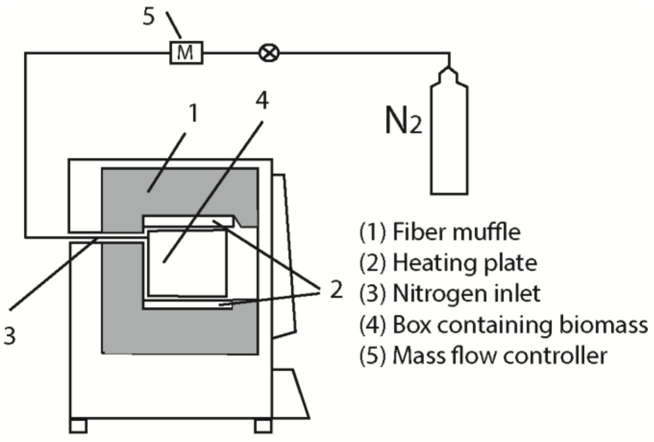

Fig. S1 The figure of the heat treatment apparatus used in this study.

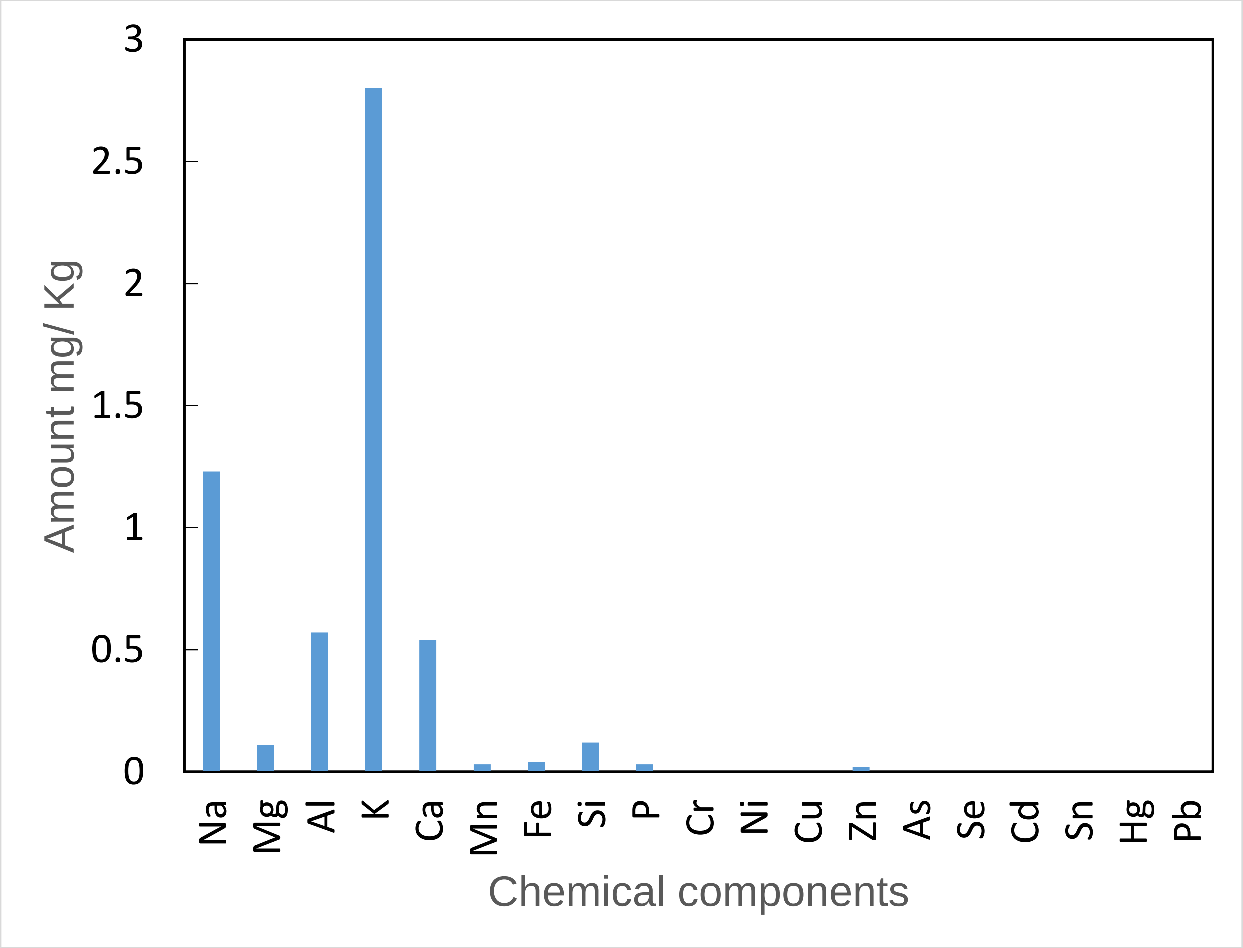

Fig. S2 Chemical compositons of the raw D. linearis fern

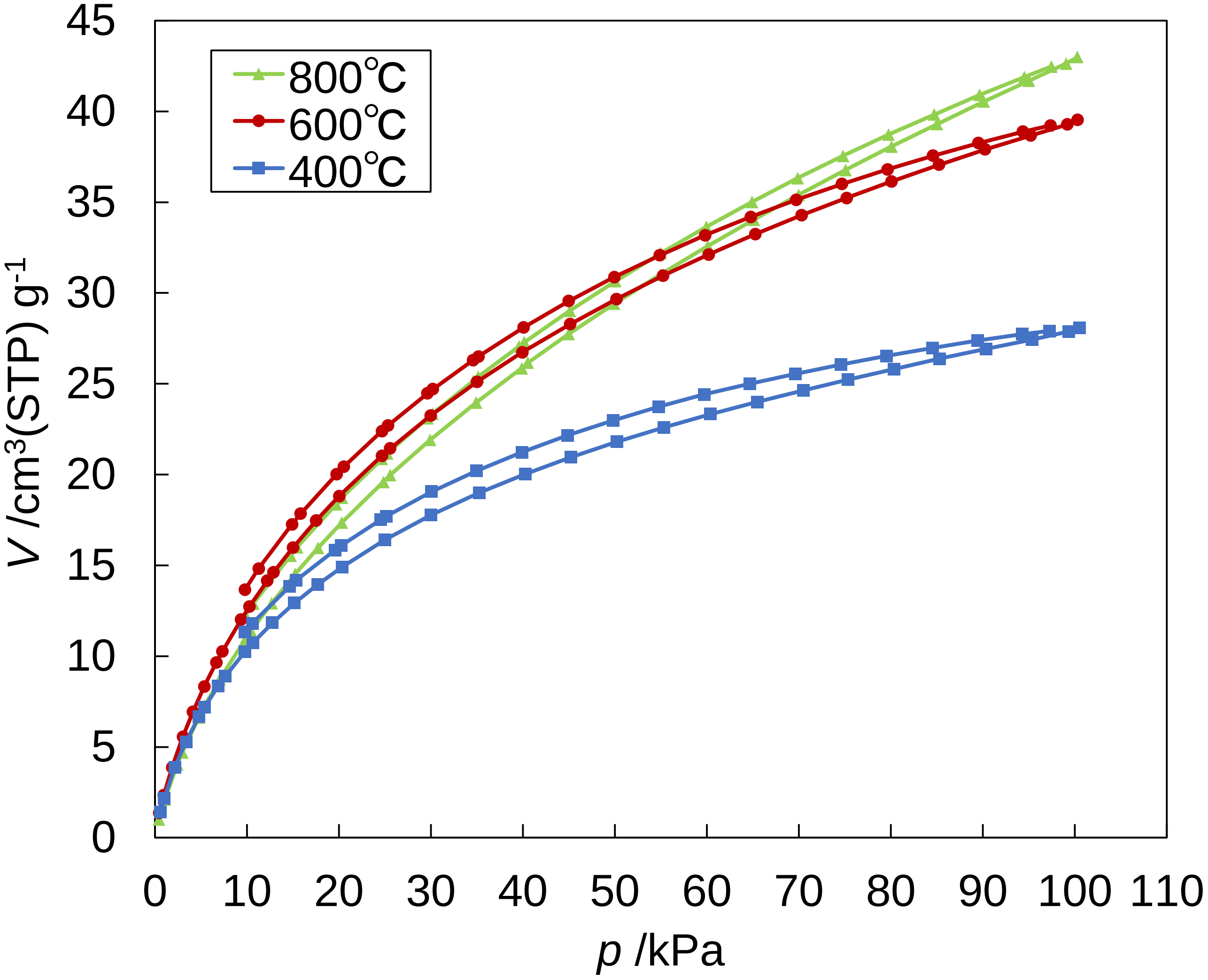

Fig. S3 CO_2 adsorption isotherms of the samples.

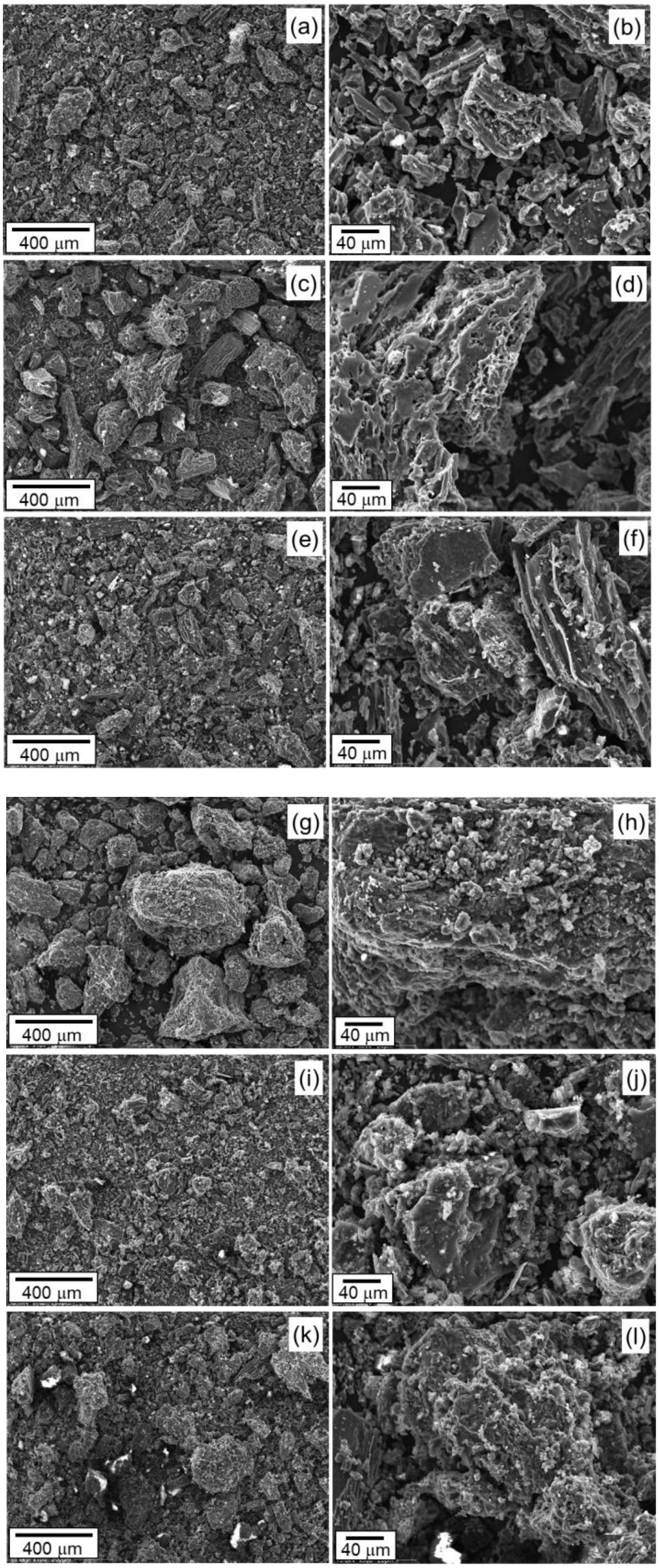

Fig. S4 SEM images of the samples activated with H_3PO_4.
(a)-(f): (raw material): (H_3PO_4) = 1:1 at the weight ratio,
(g)-(l): (raw material): (H_3PO_4) = 1:3 at the weight ratio,
(a) and (b): 400 degrees C, (c) and (d): 600 degrees C, (e) and (f): 800 degrees C,
(g) and (h): 400 degrees C, (i) and (j): 600 degrees C, (k) and (l): 800 degrees C.

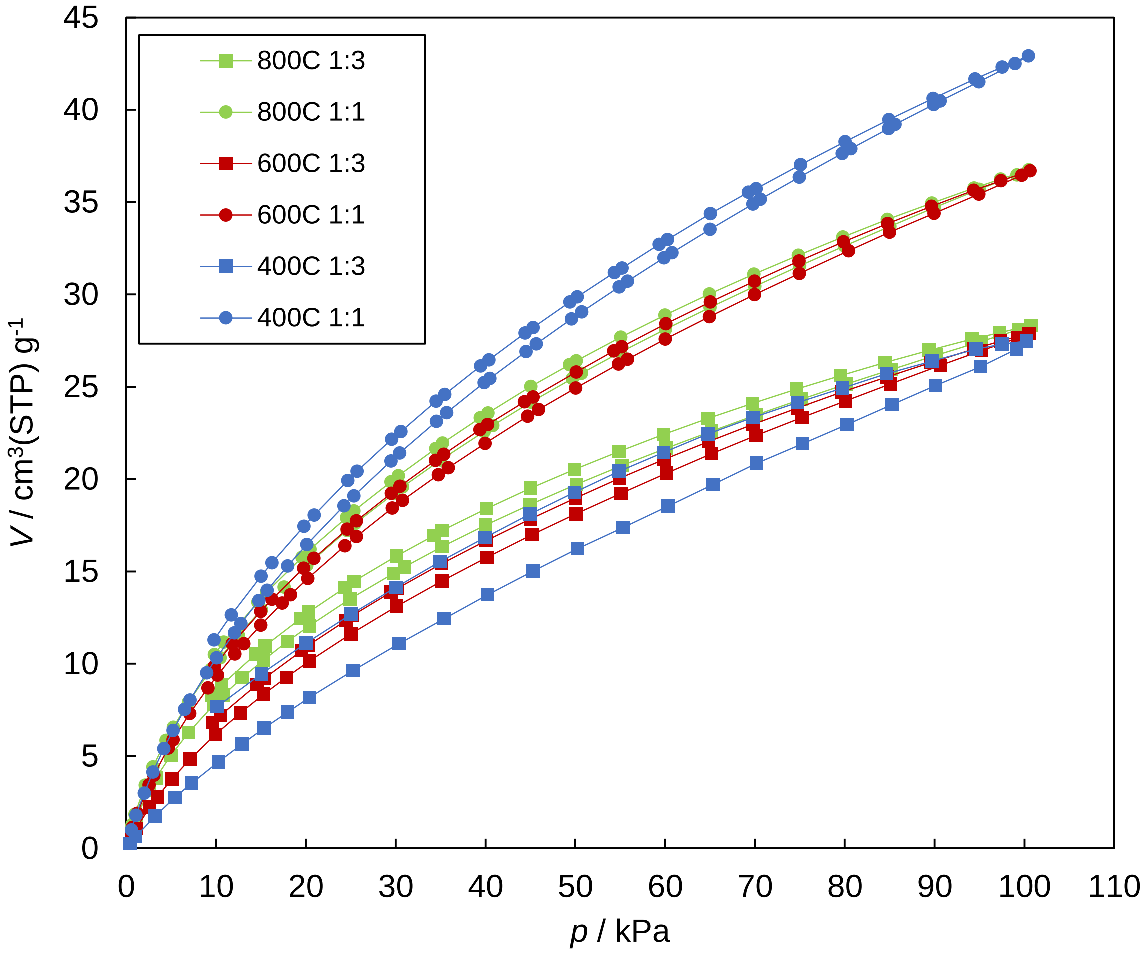

Fig. S5 CO_2 adsorption isotherms of the samples activated with H_3PO_4.

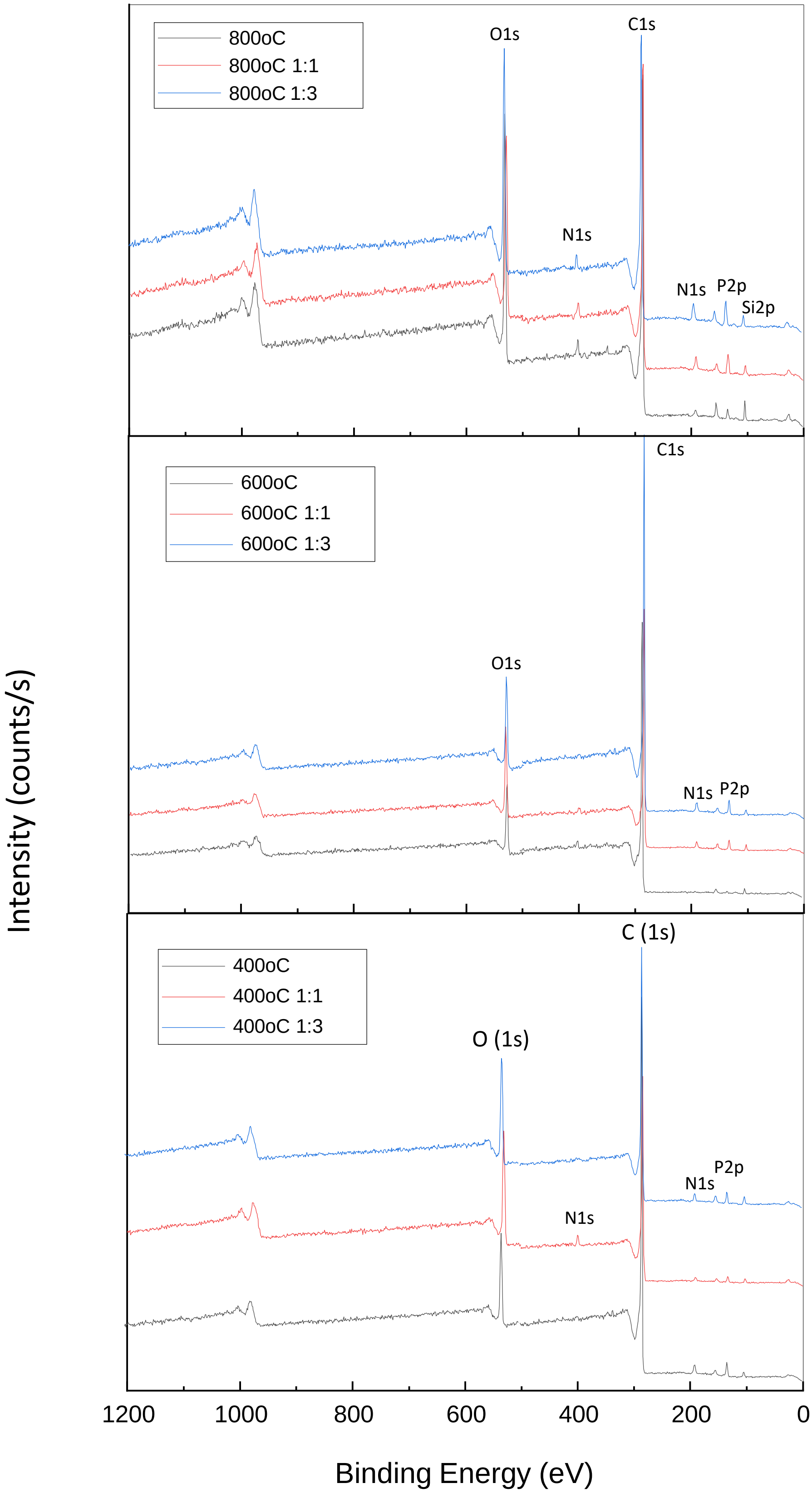

Fig S6 X-ray photoelectron spectroscopy for carbonized biochar and activated biochar at different temperatures and H_3PO_4 amounts.

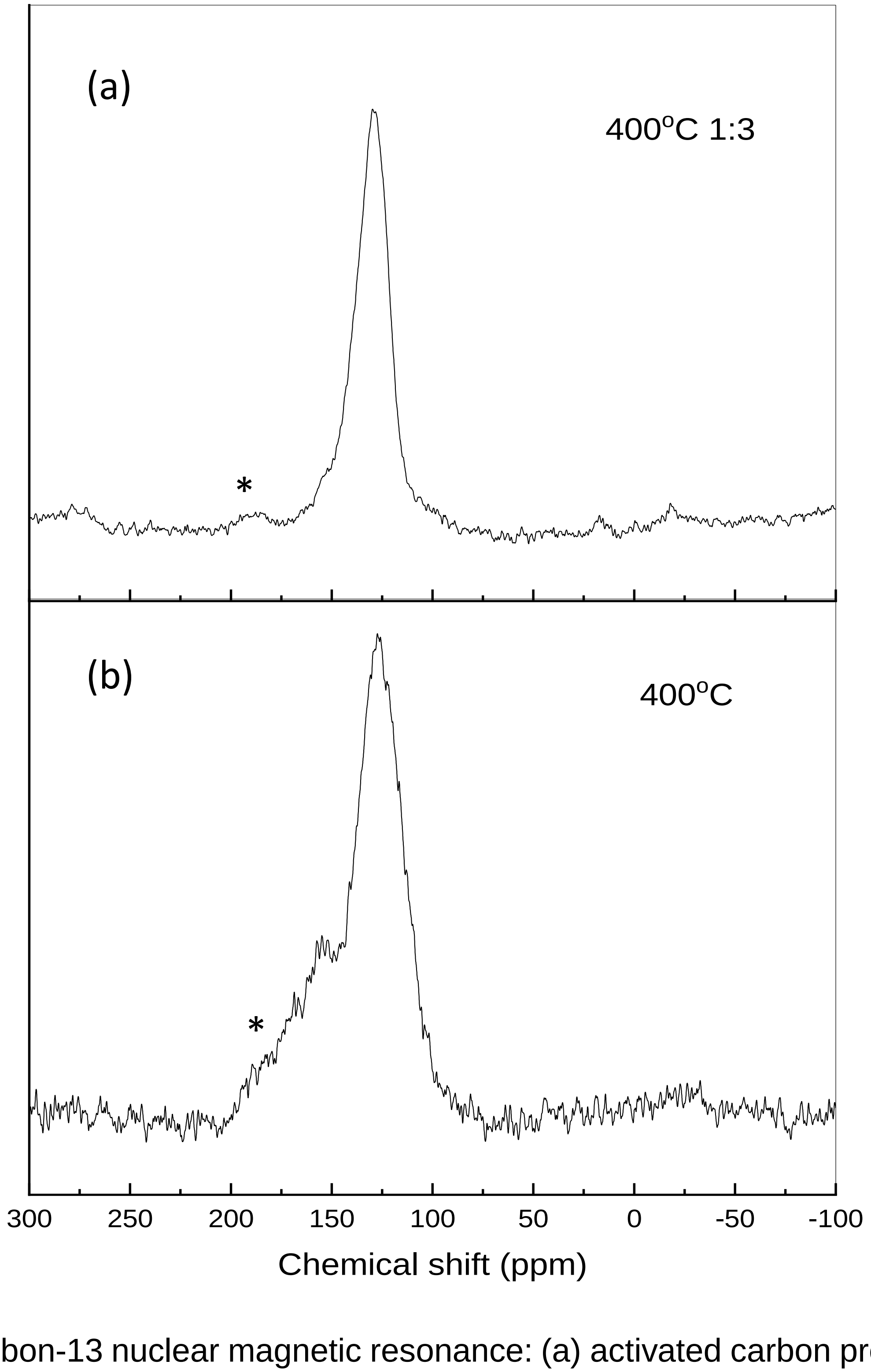

Fig. S7 Carbon-13 nuclear magnetic resonance: (a) activated carbon prepared by H_3PO_4 activation at 400oC and the mixing ratio 1:3 (w/w), (b) biochar carbonized at 400oC.

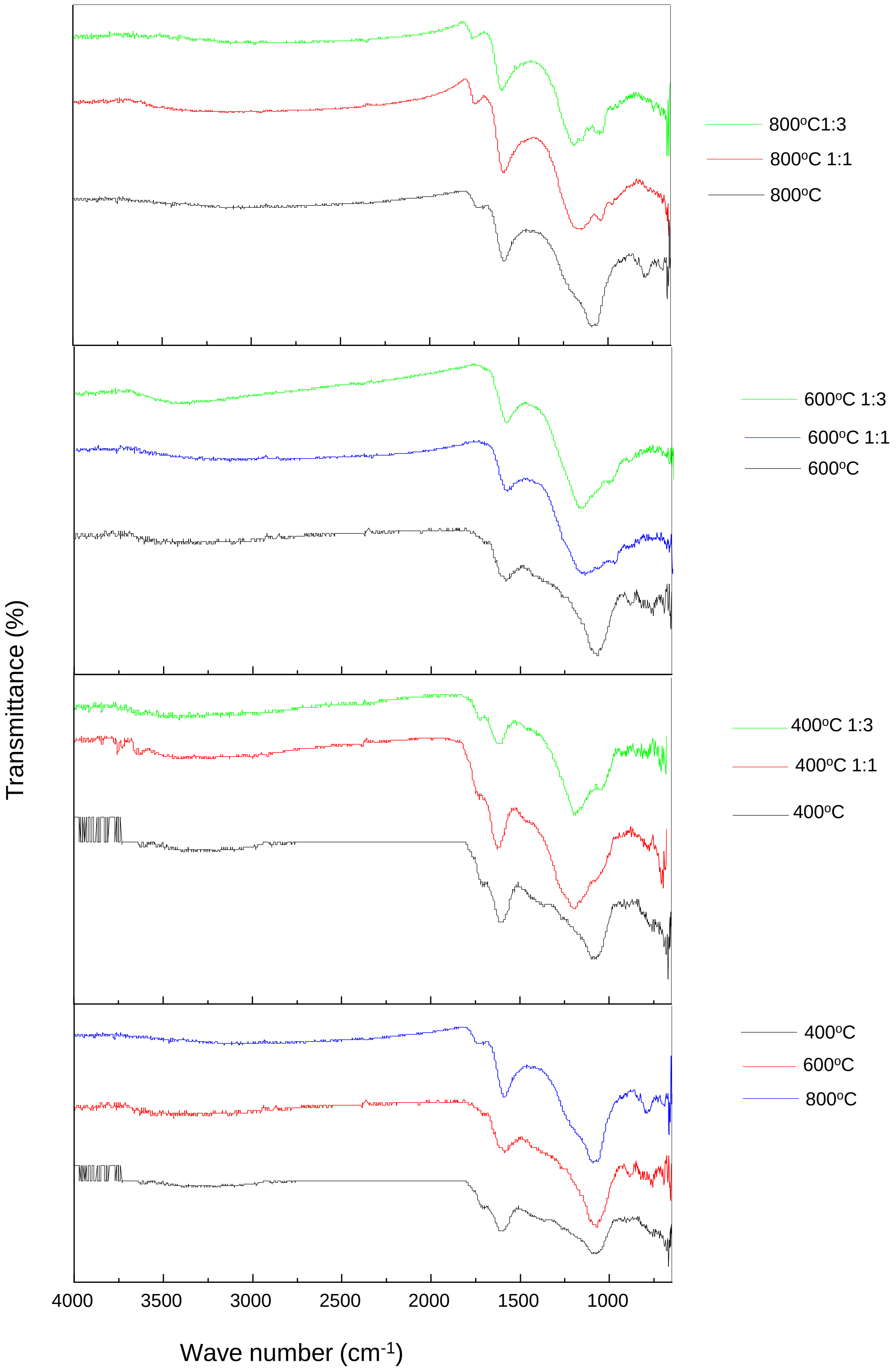

Fig. S8 Fourier transform infrared spectrophotometer at different temperatures and the mixing ratios.

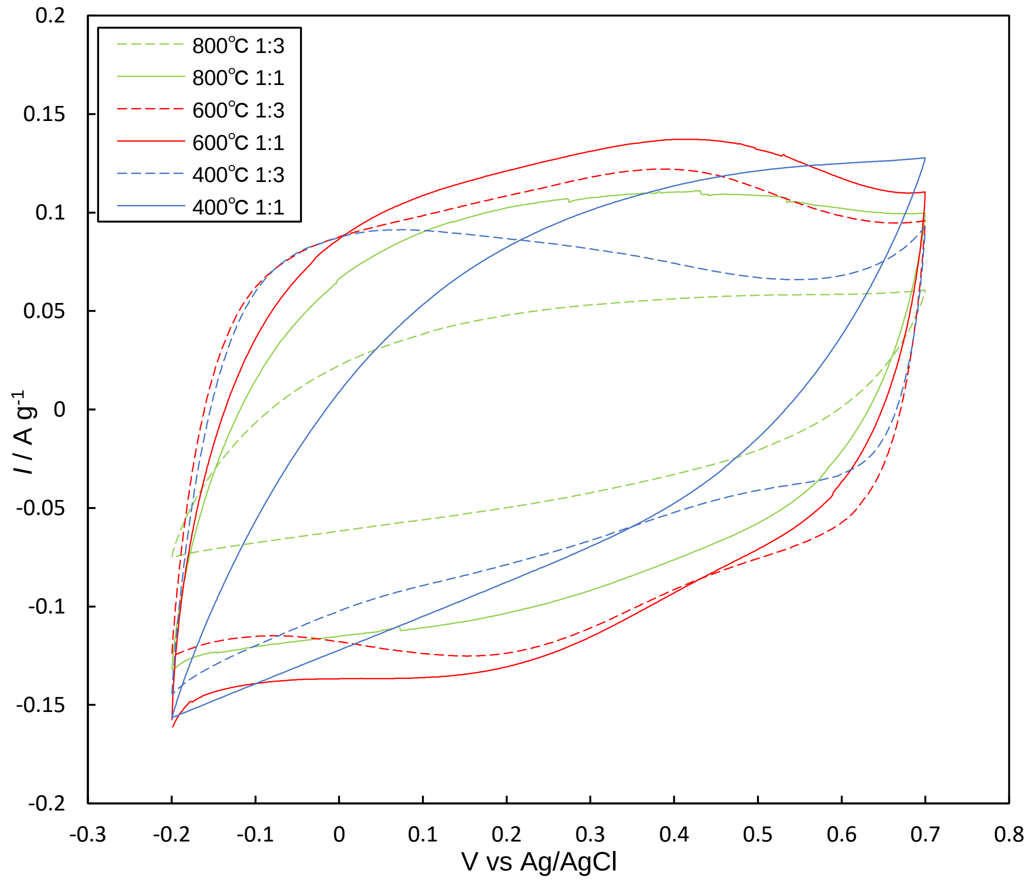

Fig. S9 The results of cyclic voltammetry of the samples activated with H_3PO_4.

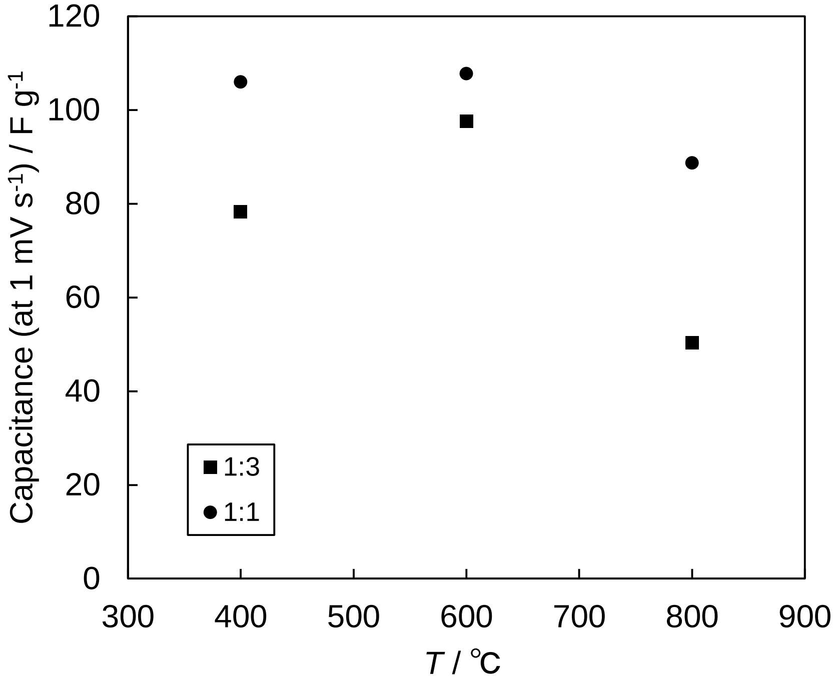

Fig. S10 The capacitance values calculated from the results of cyclic voltammetry (at 1 mV s^{-1}) of the samples activated with H_3PO_4.

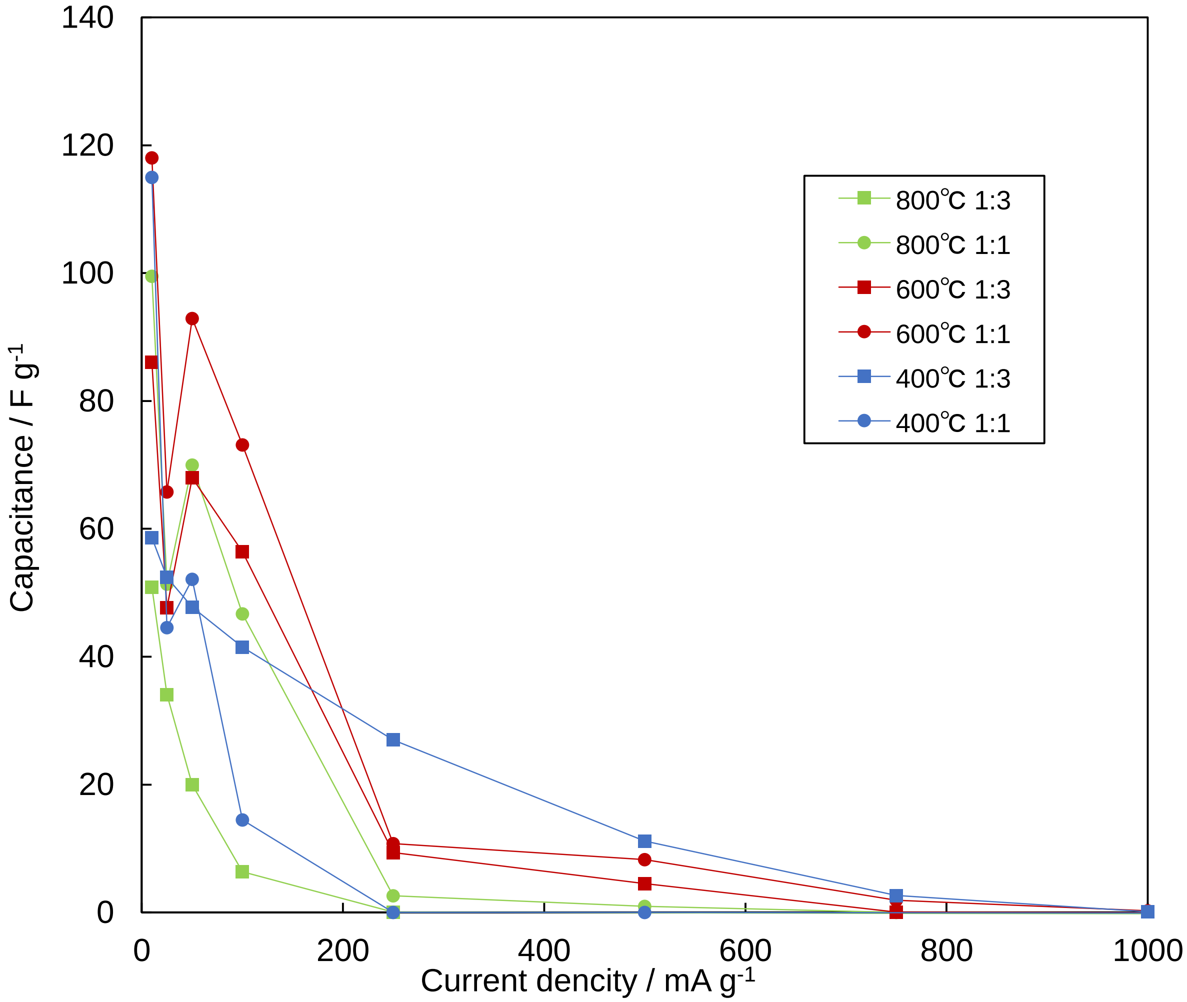

Fig. S11 The capacitance values calculated from the results of charge-discharge measurements of the samples activated with H_3PO_4.
